# Knowledge, Attitudes, and Practices of Couples Attending Premarital Clinics in the United Arab Emirates Regarding Premarital Screening and Genetic Counseling

**DOI:** 10.7759/cureus.100983

**Published:** 2026-01-07

**Authors:** Fatima A Almeleh, Amnah AlMarashdah, Batool Al Sayed Sharaf, Mariam Al Naqbi, Anoud Alkaabi, Daliah Taha

**Affiliations:** 1 Primary Healthcare Department, Emirates Health Services, Dubai, ARE; 2 Family Medicine, National Institute for Health Specialties (NIHS), Al Ain, ARE; 3 Primary Healthcare Department, Emirates Health Services, Sharjah, ARE; 4 Pediatrics, Al Qassimi Women’s and Children’s Hospital, Sharjah, ARE; 5 Primary Healthcare Department, Emirates Health Services, Khorfakkan, ARE

**Keywords:** consanguinity, genetic counseling, hereditary diseases, knowledge attitude practice, premarital screening, united arab emirates

## Abstract

Background

Premarital screening and genetic counseling are key preventive measures for reducing hereditary conditions in regions with high consanguinity, such as the United Arab Emirates. Understanding the knowledge, attitudes, and practices of couples attending premarital clinics is essential for improving service uptake and guiding targeted educational strategies.

Methodology

A multi-center, cross-sectional study was conducted from April to June 2025 across 23 premarital clinics within Emirates Health Services (EHS). Individuals aged 18 years or older who spoke Arabic or English and provided consent were included. Data were collected using a validated questionnaire administered through the EHS Data Hub. Descriptive and inferential analyses were performed to assess factors associated with knowledge, attitudes, and practices.

Results

A total of 255 participants were included; 66.7% were male, and 54.1% were aged 30-45 years. More than half were university graduates and employed. Good knowledge was reported by 77.3% of participants, positive attitudes by 92.2%, and good practice by 58.0%. Lower knowledge levels were more common among younger participants, males, and those with lower educational attainment. Females had higher odds of good knowledge (odds ratio (OR) = 2.57, 95% confidence interval (CI) = 1.26-5.27). Participants older than 45 years had higher odds of reporting good practice (OR = 4.15, 95% CI = 1.20-14.34). Good knowledge (OR = 2.27) and positive attitudes (OR = 24.09) were significant predictors of good practice.

Conclusions

Couples demonstrated strong knowledge and attitudes toward premarital screening and genetic counseling, although practice levels were comparatively lower. Targeted educational initiatives, especially for younger individuals, are needed to enhance engagement and bridge the knowledge-practice gap.

## Introduction

Premarital screening (PMS) and genetic counseling programs have emerged as fundamental public health screening programs, particularly in regions with a high incidence of genetic disorders [1]. These programs are designed to decrease the prevalence of inherited conditions by providing genetic testing and counseling as well as preventing transmission of infectious disease to couples before marriage [2]. PMS aims to prevent the transmission of infectious diseases between couples and later to their offspring. Additionally, this program ensures that couples receive appropriate health counselling and effective medical advice before marriage and gives extra care and time to individuals with positive results [3]. In the United Arab Emirates (UAE), it is compulsory for couples planning to marry in the UAE to submit a premarital medical screening certificate [4]. The UAE PMS Program includes infectious disease screening, which includes tests for the human immunodeficiency virus (HIV), hepatitis B and hepatitis C viruses (HCV and HBV), syphilis, and the rubella immunity test, which is exclusively available to females. Furthermore, tests for B-thalassemia and other significant hemoglobinopathies (HbS, HbE, HbD, HbC, HbOArab, Hb Lepore) along with ABO/RH(D) incompatibility are also performed [4]. PMS clinics also provide the hepatitis B vaccine for individuals at risk of contracting the virus and the MMR vaccine for women without rubella immunity. Additionally, the female partner should receive a human papillomavirus (HPV) vaccination, as 90% of cervical, vaginal, vulvar, anal, and penile cancers are caused by the most prevalent HPV types, which are prevented by this vaccine [5].

The burden of hereditary and genetic diseases is particularly concerning in the Arab world, where consanguinity rates are very high. In the UAE, the rate of consanguineous marriages ranges from 39% to 54.2%, with first-cousin marriages constituting 20.7% to 29.7% [6]. Additionally, there is a significant prevalence of thalassemia carriers [7]. Therefore, premarital genetic counseling and screening were introduced in certain primary care centers within the emirate’s health facilities. The expanded genetic PMS program aims to detect carriers of various recessive genetic diseases beyond hemoglobin disorders, such as sickle cell anemia and thalassemia. By identifying carriers before marriage, the program helps couples understand the risks of passing on severe genetic conditions to their offspring [8,9]. Through this initiative, individuals can receive guidance on family planning options and leverage modern technologies to reduce the incidence of these inherited diseases in future generations, ultimately promoting healthier outcomes for families and society as a whole [10]. The PMS and genetic counseling program empower couples to make informed choices about marriage, having children, and knowing any risk of developing genetic disorders. When implemented, these programs, considering the social, religious, ethnic, and cultural background of couples, have the potential to significantly reduce the incidence of high-risk marriages and the birth of affected newborns [11].

Knowledge about PMS and genetic counseling is a key determinant of couples’ compliance with these screening programs. However, existing studies have shown varying levels of awareness and knowledge about PMS and genetic counseling in different regions [8,12,13]. While awareness of the PMS program is widely recognized, there are still implementation gaps, particularly concerning the impact of genetic disorders. Couples usually have positive attitudes toward these programs, but their decisions are sometimes influenced by social and cultural constraints [8]. Given the recent introduction of genetic counseling into premarital programs, there is a lack of recent studies from the UAE addressing the knowledge, attitudes, and behaviors of couples attending premarital clinics at primary healthcare centers regarding both genetic counseling and PMS. This study aimed to assess the knowledge, attitudes, and practices of couples attending premarital clinics regarding PMS and genetic counseling, and to examine sociodemographic factors associated with variations in knowledge, attitudes, and practices.

## Materials and methods

Study design and setting

This was a multi-center, cross-sectional survey conducted between April and June 2025 across 23 centers offering premarital health services in the UAE. Six emirates were included, while Abu Dhabi was excluded as it follows a separate healthcare administration.

Sampling technique and sample size calculation

A stratified sampling approach was used to ensure proportional representation across emirates. The study population consisted of 25,553 individuals who attended premarital clinics in 2024. The sample size was calculated using a single-proportion formula with finite population correction, based on a previous Omani study reporting 84.5% positive attitudes toward PMS [14]. Using a 95% confidence level (Z = 1.96), p of 0.893, q of 0.107, and a margin of error set at 5% of p, the required sample was 184. After adjusting for a 10% non-response rate, the final target was 205 participants. Proportional allocation was applied according to the premarital clinic attendance in each emirate: Ajman (53), Dubai (43), Fujairah (24), Ras Al Khaimah (17), Sharjah (57), and Umm Al-Quwain (11).

Study participants

Eligible participants were individuals aged 18 years and above who attended premarital clinics within Emirates Health Services (EHS), fluent in Arabic or English, and who provided written informed consent. Those under 18 years old, unable to provide consent, or unable to comprehend Arabic or English were excluded.

Data collection procedure

Data collection was coordinated by the Data and Statistics Department of EHS, which oversees data management and security. Surveys were distributed through the EHS Data Hub System, the official platform for secure survey design, circulation, and storage. Invitations were sent via email and SMS, and all responses were collected anonymously to ensure confidentiality.

Knowledge, attitudes, and practices questionnaire

Participants completed a structured self-administered questionnaire developed by the research team after a literature review, with selected items adapted from previously validated instruments. The tool was reviewed for content validity by two senior researchers, translated into Arabic with back-translation for accuracy, and pretested in a pilot involving 39 participants (10% of the target sample). The final questionnaire contained 25 items across the following four sections: demographic data, knowledge, attitudes, and practices toward PMS and genetic counseling. Scoring categories were defined a priori. For the Knowledge domain (score range = 0-5), participants scoring ≥2.6 (≥50%) were classified as having good knowledge, while those scoring ≤2.5 (<50%) were considered to have limited knowledge. For the Attitudes domain (score range = 0-7), participants with scores ≥3.6 (≥50%) were categorized as having positive attitudes, while scores ≤3.5 reflected poor attitudes. For the Practice domain (score range = 0-4), scores ≥3 (≥50%) indicated good practice, while scores ≤2 indicated poor practice. Thus, a 50% cutoff was applied consistently across all domains, in line with previously published KAP studies on PMS and genetic counseling in the region [14].

Statistical analysis

Data were analyzed using SPSS version 27.0 (IBM Corp., Armonk, NY, USA). Descriptive statistics summarized demographic variables and questionnaire responses as frequencies and percentages. Associations between sociodemographic variables and knowledge, attitudes, and practices outcomes were tested using Pearson’s chi-square or Fisher’s exact test. Logistic regression was performed to identify predictors of good knowledge, attitudes, and practices, and results were reported as crude and adjusted odds ratios (ORs) with 95% confidence intervals (CIs). A p-value <0.05 was considered statistically significant.

Ethical considerations

The study was approved by the Emirates Health Services Research Ethics Committee (MOHAP/DXB-REC/D.J.J/No. 214/2024). Written informed consent was obtained from all participants before data collection. Anonymity and confidentiality were maintained, with all survey data stored securely within the EHS Data Hub System. The study adhered to the principles of the Declaration of Helsinki.

Reporting guidelines

This manuscript adheres to the Strengthening the Reporting of Observational Studies in Epidemiology (STROBE) reporting checklist for cross-sectional studies.

## Results

A total of 255 respondents were included in this study. The majority of participants were aged 30-45 years (138; 54.1%), followed by 29 years and younger (94; 36.9%) and more than 45 years (23; 9.0%). Males made up the majority of the sample (170; 66.7%). Regarding education, more than half were university/college graduates (135; 52.9%), with 65 (25.5%) being high school graduates. Nationality distribution showed 129 (50.6%) were from other nationalities, and 115 (45.1%) were UAE nationals. Most participants were employed (216; 84.7%). Regarding consanguinity, 215 (84.3%) reported no relationship, 19 (7.5%) were first-degree relatives, 16 (6.3%) had distant tribal relations, and 5 (2.0%) were second-degree relatives. For hereditary disease diagnosis, 222 (87.1%) reported no diagnosis, 17 (6.7%) were unsure, and 16 (6.3%) had a diagnosis. A family history of hereditary disease was reported by 27 (10.6%), while 26 (10.2%) were uncertain, and 202 (79.2%) had no such history (Table 1).

**Table 1:** Demographic details of included participants. Values are presented as N (%).

Variable	Category	n (%)
Age	29 years and younger	94 (36.9%)
	30–45 years	138 (54.1%)
	More than 45 years	23 (9.0%)
Gender	Male	170 (66.7%)
	Female	85 (33.3%)
Education level	Less than high school	17 (6.7%)
	High school graduate	65 (25.5%)
	University/College graduate	135 (52.9%)
	Postgraduate degree (Master’s, PhD, etc.)	38 (14.9%)
Nationality	UAE national	115 (45.1%)
	Other GCC nationalities	11 (4.3%)
	Other nationalities	129 (50.6%)
Occupation	Student	9 (3.5%)
	Unemployed	30 (11.8%)
	Employed	216 (84.7%)
Degree of consanguinity	No relationship	215 (84.3%)
	Related to the same tribe/more distant relation	16 (6.3%)
	Second-degree relatives (e.g., children of uncles/aunts)	5 (2.0%)
	First-degree relatives (e.g., cousins)	19 (7.5%)
Diagnosed with a hereditary disease	Yes	16 (6.3%)
	No	222 (87.1%)
	I do not know	17 (6.7%)
Family history of hereditary disease	Yes	27 (10.6%)
	No	202 (79.2%)
	I do not know	26 (10.2%)

Table 2 shows the distribution of knowledge, attitudes, and practices among participants. Of the 255 respondents, 197 (77.3%) demonstrated good knowledge, whereas 58 (22.7%) had limited knowledge. Attitudes were predominantly positive, with 235 (92.2%) participants reporting good attitudes compared to only 20 (7.8%) who showed poor attitudes. Regarding practice, 148 (58.0%) participants reported good practice, while a notable 107 (42.0%) reported poor practice.

**Table 2:** Knowledge, attitudes, and practice scores of the participants. Values are presented as N (%). Good and poor levels were classified using predefined scoring cutoffs.

Domain	Category	n (%)
Knowledge	Good knowledge	197 (77.3%)
	Limited knowledge	58 (22.7%)
Attitudes	Good attitudes	235 (92.2%)
	Poor attitudes	20 (7.8%)
Practices	Good practice	148 (58.0%)
	Poor practice	107 (42.0%)

Table 3 shows the distribution of knowledge levels across demographic and background variables. Younger participants (29 years and below) had a higher proportion of limited knowledge (30.9%) compared with older groups, though the difference by age was marginally significant (p = 0.051). Gender differences were more notable, as males showed a higher proportion of limited knowledge (27.6%) compared to females (12.9%), with a significant p-value (<0.001). Other variables did not meet the assumptions required for the chi-square test, as they had cells with low expected counts. Logistic regression (with males as the reference) confirmed that females had significantly higher odds of good knowledge (OR = 2.57, 95% CI = 1.26, 5.27, p = 0.01).

**Table 3:** Distribution of knowledge levels across demographic and background variables. Values are presented as N (%). Pearson’s chi-square test was performed for p-values. P-values <0.05 were considered statistically significant.

Variable	Category	Limited knowledge, n (%)	Good knowledge, n (%)	Chi-square	P-value
Age	29 years and younger	29 (30.9%)	65 (69.1%)	5.944	0.051
	30–45 years	26 (18.8%)	112 (81.2%)		
	More than 45 years	3 (13.0%)	20 (87.0%)		
Gender	Male	47 (27.6%)	123 (72.4%)	6.97	<0.001
	Female	11 (12.9%)	74 (87.1%)		
Education level	Less than high school	6 (35.3%)	11 (64.7%)	–	–
	High school graduate	20 (30.8%)	45 (69.2%)		
	University/College graduate	28 (20.7%)	107 (79.3%)		
	Postgraduate degree (Master’s, PhD, etc.)	4 (10.5%)	34 (89.5%)		
Nationality	UAE national	27 (23.5%)	88 (76.5%)	–	–
	Other GCC nationalities	4 (36.4%)	7 (63.6%)		
	Other nationalities	27 (20.9%)	102 (79.1%)		
Occupation	Unemployed	5 (16.7%)	25 (83.3%)	–	–
	Student	3 (33.3%)	6 (66.7%)		
	Employed	50 (23.1%)	166 (76.9%)		
Degree of consanguinity	No relationship	45 (20.9%)	170 (79.1%)	–	–
	First-degree relatives (e.g., cousins)	8 (42.1%)	11 (57.9%)		
	Second-degree relatives (e.g., children of uncles/aunts)	2 (40.0%)	3 (60.0%)		
	Related to the same tribe/more distant relation	3 (18.8%)	13 (81.3%)		
Diagnosed with a hereditary disease	I do not know	6 (35.3%)	11 (64.7%)	–	–
	No	49 (22.1%)	173 (77.9%)		
	Yes	3 (18.8%)	13 (81.3%)		
Family history of hereditary disease	I do not know	9 (34.6%)	17 (65.4%)	–	–
	No	48 (23.8%)	154 (76.2%)		
	Yes	1 (3.7%)	26 (96.3%)		

The majority of participants across all demographic categories demonstrated a positive attitude toward PMS (Table 4). Gender differences were minimal, with both males (91.8%) and females (92.9%) showing high levels of positive attitudes (p = 0.70). Other variables did not meet the assumptions required for the chi-square test, as they had cells with low expected counts. Younger participants reported poorer practice compared to older groups, with more than half of those aged 29 years and younger demonstrating poor practice, while individuals above 45 years old showed the highest proportion of good practice (82.6%), a statistically significant association (p = 0.01). Gender, education, nationality, occupation, degree of consanguinity, diagnosis of hereditary disease, and family history of hereditary disease did not show significant associations with practice (p > 0.05) (Table 5).

**Table 4:** Distribution of attitudes levels across demographic and background variables. Values are presented as N (%). Pearson’s chi-square test was performed for p-values. P-values <0.05 were considered statistically significant.

Variable	Category	Poor attitudes, n (%)	Good attitudes, n (%)	Chi-square	P-value
Age (years)	29 years and younger	14 (14.9%)	80 (85.1%)	—	—
	30–45 years	5 (3.6%)	133 (96.4%)		
	More than 45 years	1 (4.3%)	22 (95.7%)		
Gender	Male	14 (8.2%)	156 (91.8%)	0.109	0.70
	Female	6 (7.1%)	79 (92.9%)		
Education level	Less than high school	3 (17.6%)	14 (82.4%)	—	—
	High school graduate	6 (9.2%)	59 (90.8%)		
	University/College graduate	10 (7.4%)	125 (92.6%)		
	Postgraduate degree (Master’s, PhD, etc.)	1 (2.6%)	37 (97.4%)		
Nationality	UAE national	5 (4.3%)	110 (95.7%)	—	—
	Other GCC nationalities	2 (18.2%)	9 (81.8%)		
	Other nationalities	13 (10.1%)	116 (89.9%)		
Occupation	Student	0 (0.0%)	9 (100.0%)	—	—
	Unemployed	3 (10.0%)	27 (90.0%)		
	Employed	17 (7.9%)	199 (92.1%)		
Degree of consanguinity	No relationship	15 (7.0%)	200 (93.0%)	—	—
	First-degree relatives (e.g., cousins)	3 (15.8%)	16 (84.2%)		
	Second-degree relatives (e.g., children of uncles/aunts)	1 (20.0%)	4 (80.0%)		
	Related to the same tribe/more distant relation	1 (6.3%)	15 (93.8%)		
Diagnosed with a hereditary disease	I do not know	1 (5.9%)	16 (94.1%)	—	—
	No	17 (7.7%)	205 (92.3%)		
	Yes	2 (12.5%)	14 (87.5%)		
Family history of hereditary disease	I do not know	1 (3.8%)	25 (96.2%)	—	—
	No	17 (8.4%)	185 (91.6%)		
	Yes	2 (7.4%)	25 (92.6%)		

**Table 5:** Distribution of practice levels across demographic and background variables. Values are presented as N (%). Pearson’s chi-square test was performed for p-values. P-values <0.05 were considered statistically significant.

Variable	Category	Poor practice, n (%)	Good practice, n (%)	Chi-square	P-value
Age (years)	29 years and younger	48 (51.1%)	46 (48.9%)	9.151	0.01
	30–45 years	55 (39.9%)	83 (60.1%)		
	More than 45 years	4 (17.4%)	19 (82.6%)		
Gender	Male	74 (43.5%)	96 (56.5%)	0.51	0.47
	Female	33 (38.8%)	52 (61.2%)		
Education level	Less than high school	7 (41.2%)	10 (58.8%)	3.57	0.31
	High school graduate	31 (47.7%)	34 (52.3%)		
	University/College graduate	58 (43.0%)	77 (57.0%)		
	Postgraduate degree (Master’s, PhD, etc.)	11 (28.9%)	27 (71.1%)		
Diagnosed with a hereditary disease	I do not know	6 (35.3%)	11 (64.7%)	3.16	0.20
	No	91 (41.0%)	131 (59.0%)		
	Yes	10 (62.5%)	6 (37.5%)		
Family history of hereditary disease	I do not know	10 (38.5%)	16 (61.5%)	3.73	0.15
	No	81 (40.1%)	121 (59.9%)		
	Yes	16 (59.3%)	11 (40.7%)		

Participants with good knowledge were significantly more likely to demonstrate both good practice and positive attitudes compared with those with limited knowledge. Among individuals with limited knowledge, 36.2% reported good practice, and 77.6% had positive attitudes, whereas among those with good knowledge, 64.5% demonstrated good practice, and 96.4% had positive attitudes (p < 0.001 for both associations) (Table 6).

**Table 6:** Association of knowledge level with attitudes and practice toward premarital screening and genetic counseling. Values are presented as N (%). **: Pearson’s chi-square test for p-values. *: Fisher’s exact test for two-sided p-values. P-values <0.05 were considered statistically significant.

Knowledge level	Poor practice, n (%)	Good practice, n (%)	Chi-square	P-value	Poor attitudes, n (%)	Good attitudes, n (%)	P-value
Limited knowledge	37 (63.8%)	21 (36.2%)	14.694	<0.001**	13 (22.4%)	45 (77.6%)	<0.001*
Good knowledge	70 (35.5%)	127 (64.5%)			7 (3.6%)	190 (96.4%)	

Multivariable logistic regression analysis identified three significant predictors of good practice. Participants with good knowledge were more than twice as likely to demonstrate good practice compared to those with limited knowledge (OR = 2.27; 95% CI = 1.17-4.40; p = 0.015). Those with positive attitudes were over 20 times more likely to have good practice (OR = 24.09; 95% CI = 3.00-193.39; p = 0.003). Participants aged more than 45 years were four times more likely to engage in good practice than those aged ≤29 years (OR = 4.15; 95% CI = 1.20-14.34; p = 0.024). No other variables were significantly associated with practice after adjustment, as shown in Table 7.

**Table 7:** Multivariable logistic regression analysis of predictors of good practice regarding premarital screening and genetic counseling. Results are presented as adjusted odds ratios (ORs) with 95% confidence intervals (CIs). P-values <0.05 were considered statistically significant.

Predictor	B	OR (Exp(B))	95% CI for OR	P-value
Knowledge (good vs. limited)	0.819	2.27	1.17–4.40	0.015
Attitudes (good vs. poor)	3.182	24.09	3.00–193.39	0.003
Age >45 years (vs. ≤29 years)	1.423	4.15	1.20–14.34	0.024
Age 30–45 years (vs. ≤29 years)	0.161	1.18	0.66–2.08	0.580
Constant	–3.519	0.03	–	0.001

## Discussion

This multi-center study assessed the knowledge, attitudes, and practices of couples in the UAE regarding PMS and genetic consultation. The findings showed that the majority of participants in the present study had good knowledge of PMS (77.3%). Although conducted among university students rather than couples, a study from Abu Dhabi also reported high awareness of PMS, supporting the overall trend of increasing knowledge in the UAE [15]. However, despite the generally good knowledge documented, some critical gaps were noted in both studies. For example, in the present study, only 12.5% knew the most common hemoglobinopathy in the UAE. On the other hand, the study conducted among Abu Dhabi students reported similar knowledge gaps for less common hereditary conditions, such as glucose-6-phosphate dehydrogenase deficiency, suggesting the need for targeted educational interventions to raise awareness about these impactful genetic disorders. Internationally, studies conducted in Oman, Turkey, and other countries have also reported high levels of general knowledge about PMS. In Oman, 89.3% of adults attending primary clinics were aware of PMS [16]. Another study from Oman found that 78.1% of high-school students had heard of it [17]. In Kuwait, students from a medical background also had high PMS knowledge [18].

In contrast, awareness is lower in many African settings. A study found that only 15.4% of Ghanaian students had good PMS knowledge [19]. Another study found that only 46% of a Nigerian cohort had a good knowledge of sickle cell and PMS [20]. Regarding attitudes, the current study found that most participants expressed overwhelmingly positive views toward PMS, with 235 (92.2%) respondents reporting good attitudes. Such results reflect strong societal support for national premarital health programs and demonstrate cultural acceptance of preventive strategies in the Gulf region. Similarly, a study conducted in Oman reported that 94.3% of participants held positive attitudes, with 84.9% willing to undergo the screening test again [21]. Research in Kuwait also showed highly favorable attitudes toward PMS and genetic counseling among students [18]. Similarly, a study from Saudi Arabia among college students found that most participants had good knowledge of genetics and positive attitudes toward genetic testing [22].

Interestingly, although most participants expressed positive attitudes toward PMS, certain nuanced differences emerged. For example, some opposed laws that prohibit marriage in cases where there is a risk of genetic disease, highlighting the tension between public health policies and personal or cultural values. Cultural norms, stigma, and fear of social consequences continue to shape attitudes and influence decision-making around PMS [23,24]. In comparison, a study conducted among secondary school students in Yemen also reported generally positive attitudes yet revealed significant gaps in understanding the purpose of screening and limited readiness to accept its outcomes. These variations emphasize the role of age, education, and broader societal context in shaping attitudes toward PMS. These findings suggest the need to adapt strategies for different demographic groups [25]. Regarding practice, the study found that only 148 (58.0%) participants demonstrated good practices related to PMS, highlighting a noticeable gap between knowledge, attitudes, and actual behavior. Among university students in the UAE, a previous study also reported a gap between awareness and actual participation in PMS, although this population differs from couples attending clinics [15]. Barriers commonly reported in the literature include limited access, financial constraints, cultural resistance, and fear of stigmatization [24]. In the present study, age was significantly associated with practice. Participants over 45 years were nearly five times more likely to adopt good practices compared with those aged 29 years or younger. Similar trends have been documented in other Middle Eastern populations, where older adults tend to show higher compliance with health recommendations [26,27]. This may be attributed to greater health awareness, maturity, and personal or family experiences with hereditary diseases.

The present study found that good knowledge was associated with good practice. Furthermore, positive attitudes were an even stronger predictor of good practice, with over 23 times higher odds, which confirms the knowledge, attitudes, and practices model’s expectation that attitudes mediate the relationship between knowledge and practice. It also emphasizes that improving attitudes might have a disproportionately large impact on actual behaviors. These findings align with the broader consensus in the literature, which shows that knowledge alone is insufficient to foster practice unless accompanied by positive attitudes about the benefits and importance of PMS [28,29]. A survey of Turkish university students confirmed that higher education is correlated with a better knowledge level (p < 0.05) [29]. Similarly, in the present study, females showed significantly better knowledge than males. In another study, better knowledge was linked to higher educational attainment and positive attitudes toward genetic testing, and the pattern of educational influence on knowledge is a common thread across regionally and culturally similar populations [28]. The findings of the current study underscore the critical role of PMS programs as public health interventions in the UAE and similar countries, but cultural and religious debates initially influenced their adoption and acceptance, mirroring the nuanced attitudes found in the present UAE study [30].

This study has several strengths, including its multi-center design, proportional sampling across six emirates, and use of a validated bilingual questionnaire. However, certain limitations should be acknowledged. First, the cross-sectional design precludes causal inferences between knowledge, attitudes, and practices. Second, reliance on self-reported data may introduce recall and social desirability bias. Third, the study population was limited to couples attending EHS clinics, which may reduce generalizability to other healthcare settings in the UAE. Finally, some subgroup analyses were underpowered due to small sample sizes.

## Conclusions

The findings of the present study show that while most participants demonstrated good knowledge and overwhelmingly positive attitudes toward PMS, only a little more than half translated these into good practice. Knowledge and attitudes were found to be strong predictors of practice, which emphasizes the importance of educational awareness and counseling initiatives. Younger age, male gender, and lower educational attainment were associated with limited knowledge, suggesting that targeted interventions for these groups may help improve uptake. The high prevalence of positive attitudes, regardless of demographic background, reflects strong community support for PMS programs. However, the observed gap between knowledge and actual practice highlights persistent barriers that need to be addressed. These may include cultural beliefs, misconceptions about genetic risk, or a lack of perceived personal relevance. Despite the findings of the present study, studies with a larger sample size should be conducted to validate these findings. Integrating genetic counseling more systematically into PMS, alongside culturally sensitive education, may enhance program uptake and reduce the burden of hereditary disease.

## Appendices

 Study questionnaire

**Table 8:** Premarital screening and genetic counseling questionnaire.

Item number	Question/Statement	Response option 1	Response option 2	Response option 3	Response option 4	Response option 5
1	Age	29 years and younger	30–45 years	>45 years	—	—
2	Gender	Male	Female	—	—	—
3	Level of education	Less than high school	High school graduate	University/College graduate	Postgraduate degree	—
4	Nationality	UAE national	Other GCC nationalities	Other nationalities	—	—
5	Occupation	Student	Employed	Unemployed	—	—
6	Degree of consanguinity	First-degree relatives	Second-degree relatives	Same tribe/Distant relation	No relationship	—
7	Diagnosed with a hereditary disease	Yes	No	I do not know	—	—
8	Family history of hereditary disease	Yes	No	I do not know	—	—
9	Tests in premarital screening	Genetic blood disorders	Infectious + genetic blood disorders	Infectious diseases	—	—
10	Screening decreases hereditary diseases	Yes	No	I do not know	—	—
11	Consanguinity increases disease risk	Yes	No	I do not know	—	—
12	The most common hemoglobinopathy in the UAE	Thalassemia carrier	Thalassemia disease	Sickle cell carrier	Sickle cell disease	I do not know
13	Hepatitis B transmission mode	Partner to partner	Mother to fetus	Blood/Products	All of the above	—
14	Importance of premarital screening	Very important	Important	Neutral	Not important	Not important at all
15	Genetic counseling aids decisions	Strongly agree	Agree	Neutral	Disagree	Strongly disagree
16	The law should restrict high‑risk marriages	Strongly agree	Agree	Neutral	Disagree	Strongly disagree
17	Cultural beliefs affect attitude	Strongly agree	Agree	Neutral	Disagree	Strongly disagree
18	Genetic testing benefits society	Strongly agree	Agree	Neutral	Disagree	Strongly disagree
19	STD screening necessary	Strongly agree	Agree	Neutral	Disagree	Strongly disagree
20	Response to the risk of the affected child	Continue marriage (faith)	Depends on probability	Cancel engagement	Emotional reasons	Family pressure/Unsure
21	Took preventive measures	Yes	No	No measures needed	—	—
22	Emotional challenges	Yes	No	—	—	—
23	Awareness should start in school	Strongly agree	Agree	Neutral	Disagree	Strongly disagree
24	Marriage against medical advice is wrong	Strongly agree	Agree	Neutral	Disagree	Strongly disagree
